# The Dermatologist’s Approach to Onychomycosis

**DOI:** 10.3390/jof1020173

**Published:** 2015-08-19

**Authors:** Jenna N. Queller, Neal Bhatia

**Affiliations:** 1 Dermatology Chief Resident at Harbor-UCLA Medical Center, Torrance, CA 90502, USA; E-Mail: Jenna.Queller@gmail.com; 2 Director of Clinical Dermatology, Therapeutics Clinical Research, San Diego, CA 92123, USA

**Keywords:** onychomycosis, onycholysis, subungual hyperkeratosis, recurrence, maintenance

## Abstract

Onychomycosis is a fungal infection of the toenails or fingernails that can involve any component of the nail unit, including the matrix, bed, and plate. It is a common disorder that may be a reservoir for infection resulting in significant medical problems. Moreover, onychomycosis can have a substantial influence on one’s quality of life. An understanding of the disorder and updated management is important for all health care professionals. Aside from reducing quality of life, sequelae of the disease may include pain and disfigurement, possibly leading to more serious physical and occupational limitations. Dermatologists, Podiatrists, and other clinicians who treat onychomycosis are now entering a new era when considering treatment options—topical modalities are proving more effective than those of the past. The once sought after concept of viable, effective, well-tolerated, and still easy-to-use monotherapy alternatives to oral therapy treatments for onychomycosis is now within reach given recent study data. In addition, these therapies may also find a role in combination and maintenance therapy; in order to treat the entire disease the practitioner needs to optimize these topical agents as sustained therapy after initial clearance to reduce recurrence or re-infection given the nature of the disease.

## 1. Introduction

The diagnosis and treatment of onychomycosis has entered a new era, which in some ways is trivial due to the ease of “detection” by way of the internet and media, patients can deduce the diagnosis before it has been clinically proven. Yet in the same breadth clinicians are encouraged since topical therapies which were once thought to be ineffective now have been proven more effective and safe in multiple clinical trials. As the mechanisms of therapy have become more elucidated, so has the success rate which encourages both practitioners and patients to adhere to therapy despite the time needed to achieve that once elusive clearance of disease. Moreover, as the mechanisms of therapy are better understood and consolidated into potential treatment regiments there will be more study data and clinical experience necessary to dictate the utility of combinations between topical therapies and systemic treatments, in addition to topical keratolytics, concomitant use of nail polish, and the optimization in patients with diabetes mellitus, peripheral vascular diseases, and other comorbities.

Unfortunately, as medicine continues during the era of patients making diagnoses from the internet and the photos driven by the media direct to consumer advertising, the perception of onychomycosis as a disease that requires objective proof of diagnosis and aggressive therapies continues to be an obstacle for successful treatment. The presence of any nail discoloration or change in the integrity leads to a presumptive diagnosis of onychomycosis to the untrained eye (Table 1).

**Table 1:** Mimickers of Onychomycosis.

Mimickers of Onychomycosis	Reference
Psoriasis	[1]
Lichen Planus	
Bacterial Infections	
Onychogryphosis	
Traumatic onychodystrophies	
Yellow Nail Syndrome	
Toenail Cellulitis	
Contact Dermatitis	
Nail-bed Tumors	
Onycholysis, nonspecific	
Pachyonychia Congenita	

## 2. Terminology

The appropriate diagnostic terminology is necessary when documenting the clinical findings of onychomycosis, not only to convey the objective assessment, but also as a marker of therapeutic milestones for improvement. Charting of the presentation is often difficult when there is a history of trauma to the nails, the chronicity of disease has obscured normal markers, and other nail disorders are present. The correct application of diagnostic terminology will convey an accurate presentation. Onychomycosis is a fungal infection of the nail unit [1]. Subungual thickening or hyperkeratosis can occur under the nail plate, resulting in onycholysis or lifting of the nail bed. Onycholysis, specifically is the loss of plate-bed adhesion. Onycholysis does not by itself signify onychomycosis. It can be seen in many other diseases such as psoriasis and lichen planus [1]. There are numerous potential causes such as irritants or trauma, infections (*Candidiasis*, *Syphilis*), and drug-induced cases, which often affect multiple nails [1].

Onychomycosis more commonly involves the toenails [1]. It is caused by a variety of fungi including dermatophytes, non-dermatophyte molds, and *Candida* [2,3]. More specifically, there are five subtypes related to the method of fungal invasion of the nail unit, the most common being distal lateral subungual onychomycosis (DLSO) (Table 2) [4,5]. In DLSO, the fungus enters the distal lateral part of the nail bed, the region of the hyponychium, often as an extension of tinea pedis. Hyperkeratosis occurs under the nail plate, resulting in onycholysis, with subungual thickening. White superficial onychomycosis is less common than DLSO, accounting for about 10% of onychomycosis cases [6]. The superficial nail plate is usually involved initially; most commonly caused by *Trichophyton mentagrophytes* and several non-dermatophyte molds (such as *Fusarium*, *Aspergillus*, and *Acremonium* spp.) [7]. To the patient the nail feels coarse but may become soft and crumbly where the fungus has initiated the infection [8], and it can be scraped off easily with a scalpel [7]. Proximal subungual onychomycosis (PSO), a relatively uncommon subtype, occurs when the fungus invades under the cuticle or nail plate, and advances from the proximal to distal part of the nail [5]. Endonyx onychomycosis differs from DLSO because of the absence of nail-bed hyperkeratosis and onycholysis, and is usually caused by *Trichophyton soudanense* [7] (not found in the United States). *Candida* onychomycosis only affects immunosuppressed patients and the presentation involves the entire nail plate, often with paronychia [7].

**Table 2:** Subtypes of Onychomycosis.

Subtypes of Onychomycosis	Reference
Distal lateral subungual onychomycosis (DLSO)	[1]
White superficial onychomycosis (WSO)	
Proximal subungual onychomycosis (PSO)	
Endonyx onychomycosis (EO)	
Candidal onychomycosis	

## 3. Prevalence and Risk Factors

The prevalence of onychomycosis is increasing; according to studies over the last 20 years, it has increased from 2% to 14% [7], especially with a rise in men and the elderly [9,10], but is relatively uncommon in children [11]. Psoriasis is also a risk factor, particularly in dermatophyte onychomycosis [12]. A 27% prevalence of onychomycosis was reported in psoriatic patients when the toenail was clinically abnormal, and 13% onychomycosis was found in psoriatic patients overall [13].

## 4. Diagnosis

A definitive diagnosis of onychomycosis is made by the presence or absence of fungal elements using potassium hydroxide (KOH) preparation or a periodic acid-Schiff (PAS) stain, and identification of the fungi with a culture. Most experts perform a KOH and do a fungal culture. Many dermatologists use a PAS stain because it is less subjective to errors than fungal cultures; however it may be more expensive. Only about 50% of dystrophic nails are attributed to fungi, the rest are a feature of something else such as trauma, psoriasis, or onychogryphosis [7]. There are varying techniques when sampling the different subtypes of onychomycosis. In DLSO, the specimen should be obtained from the nail bed by curettage to maximize the yield for study. Removing the onycholytic nail plate will yield a more useful sample at a site most proximal to the cuticle, since that is where the highest concentration of hyphae are located. In PSO, clinicians should pare down the overlying nail plate before sampling the ventral plate to obtain the optimal exposure. In WSO, a 15 blade is often used to effectively remove a specimen from the nail surface. In Candidal onychomycosis, specimens should be sampled from the most proximal and lateral edges.

## 5. Determining Severity and Outcomes

In 2011, Carney *et al.* proposed a classification system for grading the severity of onychomycosis [14]. The authors’ goals were to establish an objective method for defining mild, moderate and severe onychomycosis using a numerical scoring system. This scoring classification accounts for the area of involvement (range, 0–5), which is then multiplied by the score for the proximity of disease to the nail matrix (range,1–5), and 10 points are added if the presence of a longitudinal streak or patch is seen or if there is greater than 2 mm of subungual hyperkeratosis. Mild nail involvement with onychomycosis is classified as a score of 5 or less; moderate, 6 through 15; and severe, 16 through 35. A baseline or clinically cured nail is classified as a score of 0. This index provides a standardized method for evaluating onychomycosis that can be utilized throughout a patient’s treatment course. This is a promising tool, however, further research is necessary in order to properly correlate nail disease severity with prognostic outcomes.

## 6. To Treat or Not to Treat?

Onychomycosis is notoriously difficult to treat. The main goals of onychomycosis treatment include eradication of pathogens, restoration of healthy nails, and prevention of relapse or recurrence. It is an infectious disease that deserves prompt and appropriate care [4]. Successful management of onychomycosis can be challenging due to the limited availability of effective treatments, patient adherence, and recurrence or reinfection [15]. The disease is often associated with substantial distress, which can affect the patient’s quality of life. Aside from reducing quality of life, the disease sequelae can include pain and disfigurement, in addition to serious physical and occupational limitations resulting in disabilities [16,17]. There is also a high potential for dissemination to other nails and local skin. Complications do arise in immunocompromised patients, as well as those with Diabetes Mellitus. In general, most clinicians feel that onychomycosis is an important problem that should be properly diagnosed and treated, especially if it is symptomatic or bothersome. Patients often present to the dermatologist with a long history of onychomycosis; substantial nail involvement, and therefore usually require oral therapy. The addition of an effective topical antifungal to the physician’s therapeutic armamentarium would address an important unmet medical need. In the past, when encountering a patient with onychomycosis who was not a candidate for oral antifungals, most clinicians were left with no adequate treatment modalities. However, multiple clinical trials now have demonstrated that there are viable topical treatments. As of today, treatment options include systemic agents, topical agents, and laser procedures.

## 7. Systemic Therapies

Two systemic treatments, terbinafine and itraconazole, are approved by the US Food and Drug Administration (FDA) for onychomycosis, taken orally for three months, with lab monitoring every six weeks [18]. Pulsed itraconazole is only approved for the treatment of fingernail onychomycosis, and fluconazole is not FDA-approved at all for this indication [19].

When deciding between systemic or topical therapies, several factors must be considered. One should take into account if there is lunular or matrix involvement, the overall severity (number of nail involvement), the patient’s risk factors, (hepatic, cardiac, *etc.*), concomitant medications and potential for CYP450 drug interactions, lifestyle choices with alcohol intake, patient reliability for laboratory, follow-up and monitoring, and of course patient preference, concerns, and fears. Additionally, assessment of one’s risk of resistance to antifungal therapy should be considered, as *Fusarium* spp. and other non-dermatophyte filamentous fungi are especially difficult to eradicate using standard treatment with terbinafine and itraconazole [20] PCR fungal identification can aid in demonstrating the presence of molds in order to use an alternative treatment [20].

## 8. New Topical Therapies

The development of topical antifungals focusing on new formulations of existing antifungals and formulating new agents entirely has been ongoing in the recent years since there is an obvious need for an effective topical modality. The use of topical ciclopirox 8% lacquer was disappointing both in clinical trials and in practice given the low complete clearance percentages [21,22]. Poor nail penetration limited its effectiveness, and as a result topical ciclopirox has been reserved for mild cases of the disease [23]. The reported mycological cure rates for ciclopirox topical lacquer are 29%–54.3% [21,24]; and complete cure rates by proof of a negative culture and negative KOH combined with investigator assessments are 5.5%–8.5% [21].

The new topical formulations seem to be very promising, thanks to new technology in the vehicles as well as optimal application of the active antifungal ingredients. The route of entry into the nail plate and the nail bed plays a vital role in determining the efficacy of a drug. Oral agents reach the nail bed by achieving antifungal levels in the blood stream that are in excess of the minimum inhibitory concentration. The primary route of drug delivery for topical lacquers is transungual, with the agent applied to the dorsal aspect of the nail plate and it then penetrates to the underlying nail bed. The new topical agents approved in the US for the treatment of onychomycosis are solutions with increased nail penetration characteristics and low surface tension; therefore, these agents penetrate via the transungual route, and through the space between the nail plate and the nail bed [25]. This low surface tension is believed to enhance penetration and achieve clinical success by providing a dual mode of delivery in accessing the nail bed [26]. This route is an essential means for its drug delivery by circumventing the thickness of the nail plate.

Efinaconazole’s primary mechanism of action is blockage of ergosterol biosynthesis, through sterol 14 α-demethylase inhibition [27,28]. Efinaconazole has a broad spectrum of activity against dermatophytes, non-dermatophytes, and yeasts. It works against the most common pathogens including, *T. rubrum* and *T. mentagrophytes* and *C. albicans*, and was found to be more potent than previously available antifungal agents [27,29].

Elewski *et al.* conducted a single-day study including 11 subjects with onychomycosis [30]. They evaluated the ability of efinaconazole 10% topical solution with fluorescein in its vehicle in order to demonstrate its spread into the subungual space between the nail plate and nail bed after application to the distal end of the toenail. The advantages of this study are the photographs they published that exhibit the location of the medication under UV light. Evaluations under both visible and UV light indicated that the vehicle had reached the subungual space, with deposition of fluorescein wherever vehicle had spread, including in the nail bed.

Evaluation of concurrent use of nail polish in cadavers has been reported, most importantly revealing radiolabeled permeation of efinaconazole with minimal disruption to the integrity of the polish. Further phase IV studies with topical agents in combination with nail polish are necessary to determine long-term efficacy while giving patients options for cosmetic concealment of their dystrophic nails. Nevertheless, this study demonstrates that nail polish does not appear to effect efinaconazole 10% topical solution [31].

In 2013, two pivotal multi-centered, randomized double-blind vehicle controlled trials were performed over 52 weeks and included 1655 participants with toenail distal lateral subungual onychomycosis [32]. Patients were randomized (3:1) to efinaconazole or vehicle group and applied it once daily for 48 weeks, with a four-week post treatment follow-up visit. The primary endpoint of the study was a complete cure, with no clinical involvement of the target toenail, in addition to a negative potassium hydroxide examination and negative fungal culture at week 52. Mycological cure rate was 56%; significantly greater than vehicle. At the 52-week follow-up visit, nearly half of the subjects in both studies had treatment success with efinaconazole, shown by 90% toenail clearance in nearly 50% of subjects. Moreover, in contrast to previous studies with ciclopirox, efficacy was not dependent upon daily debridement.

Tavaborole represents a new class of antifungals that consist of protein synthesis inhibitors that exhibit antifungal properties [33]. Tavaborole is a novel, boron-based topical agent approved for the treatment of onychomycosis caused by *T. rubrum* and *T. mentagrophytes* [34]. Tavaborole inhibits leucyl-tRNA-synthetase, resulting in inhibition of fungal protein synthesis and extinction of fungal cell growth [35]. In two phase-III trials in patients with mild-to-moderate DLSO affecting 20% to 60% of a target great toenail were randomized 2:1 to tavaborole or vehicle once daily for 48 weeks. They found rates of negative mycology (31.1%–35.9% *vs.* 7.2%–12.2%) and complete cure (6.5% and 9.1% *vs.* 0.5% and 1.5%), therefore rates were significantly better than vehicle after 48 weeks of treatment [36]. Tavaborole was also found to penetrate through the nail plate approximately 250 times greater than ciclopirox [37]. The overall amount of Tavaborole that penetrated through the nail in the 14-day period amounted to 16% of the applied dose compared to 0.1% for ciclopirox.

## 9. Laser and Light Devices

### 9.1. Nd:YAG

The use of lasers has increased as a means of treating onychomycosis without the side effects of systemic therapy. A 1064 neodymium-doped yttrium aluminum garnet (Nd:YAG) laser called Pinpointe^®^ Foot Laser (Cynosure, Westford, MA, USA), is marketed for the treatment of onychomycosis. It is understood that due to its longer wavelength, the 1064-nm Nd:YAG is able to deeply penetrate tissue and efficiently target fungal overgrowth in the nail bed [38].

Unfortunately, *in vitro* effects of Nd:YAG laser systems on fungal growth have yielded differing results. One study showed that seven out of eight (87.5%) patients treated with a total of two to three treatments spaced three weeks apart (223 J/cm^2^, 0.65 ms, 2-mm spot size, two passes), obtained a negative culture after two to three sessions [39]. Another study yielded less striking results with only 9.3% of the nails treated achieving a complete cure [40]. In comparison, Kimura and colleagues treated 13 patients (37 total nails) with two to three sessions spaced four and eight weeks apart with 51% having microscopically negative complete clearance at the six-month follow-up [41].

### 9.2. Carbon Dioxide Laser

The carbon dioxide (CO_2_) laser was one of the first and oldest laser therapies employed to treat onychomycosis, relying on ablation to treat onychomycosis. While the studies were promising when they were first released, the advent of less invasive laser treatment options has made the CO_2_ laser a less favorable treatment option for onychomycosis. CO_2_ lasers are known to cause pain and penetrates deep into tissues, which can lead to scarring and disfigurement. Apfelberg *et al.* in 1984 reported nine cases of onychomycosis treated with CO2 laser therapy [42]. Years later, Borovoy *et al.* treated 200 patients with culture-confirmed onychomycosis with one CO_2_ laser session followed by 12–18 months of topical antifungal cream [43]. Nails were debulked prior to the laser treatment and then patients were instructed to file down their nails regularly with a single-use emery board. They reported 75% total clearance without recurrence with three years of follow-up.

### 9.3. UV Light Therapy

It is known that UV light is highly germicidal, therefore that led researchers to study the possible therapeutic applications of UV light in treating onychomycosis [44]. In 2008, Dai and colleagues studied *in vitro* susceptibility and resistance of *T. rubrum* with UVC (254 ± 2 nm) light [45]. After incubation of human nail fragments with *T. rubrum* for four weeks, dermatophyte clearance was seen in 50% of samples with 36 J/cm^2^ UVC, in 80% of samples with 72 J/cm^2^, and in 100% of samples with 144 J/cm^2^ [42]. In addition, *T. rubrum* did not show any resistance to the UVC spectrum. These researchers suggested protecting adjacent tissues with UV blockers in order to address the mutagenic nature of UV light in normal tissue. In 2013, Chronin *et al.* found conflicting results [43]. The study demonstrated that treatment with UVC was not feasible, as UV light would not penetrate the nail until 320nm and wavelengths of 320 nm or greater would not eradicate *T. rubrum* [46].

### 9.4. Photodynamic Therapy

Photodynamic therapy (PDT) has been a very widely investigated method for the treatment of onychomycosis, which is a combination of a photosensitizing agent followed by irradiation with specific wavelength of light, thus enabling maximal absorption of light by the desired target. *T. rubrum* metabolizes aminolevulinic acid to protoporphyrin IX which then can result in a 50% growth reduction. When using PDT for onychomycosis, the organism absorbs the photosensitizing agent, making it more susceptible to destruction than surrounding healthy tissue. While many photosensitizers have been evaluated for this purpose, 5-aminolevulinic acid (5-ALA) is the most frequently studied.

A clinical trial evaluating PDT for treatment of onychomycosis did so after chemical avulsion of the toenail [47]. After chemical avulsion with urea, the nail bed was treated with 20% 5-ALA for 3 h, followed by red light. After three sessions, authors reported a 43.3% clinical and mycological cure rate at 12-month follow-up [47]. Similarly, Piraccini *et al.* reported a case of onychomycosis successfully treated after chemical avulsion and PDT with negative mycological and clinical assessment after two years of follow-up [48].

### 9.5. Dual Wavelength 870-/930-nm Laser

Bornstein and colleagues found that 4074 J/cm^2^ of 870/930 nm resulted in 100% eradication of bacteria, fungi, and yeast [49]. The researchers treated patients with four sessions of the 870-/930-nm laser followed by the 930-nm laser alone. After 60 days, clear nail growth was observed in four out of seven patients, and all nail cultures were negative [44]. The investigators measured a decrease in trans-membrane potentials and an increase in reactive oxygen species (ROS) generated in MRSA and *C. albicans*. No observable damage to the nail matrix was observed, but photodamage to the pathogens was achieved at physiologic temperatures. The selective feature presents the possibility for future employment in human cutaneous antimicrobial therapy.

Another trial confirmed the efficacy of the settings and found both negative cultures and at least 3 mm of clear nail growth in four months in 39% of nails after the last treatment [50]. When evaluated at nine-month follow-up, 38% of patients maintained negative culture and microscopy [51].

## 10. Managing Expectations for Increased Patient Satisfaction

Guidance strategies and a clear discussion of expectations can significantly impact long-term outcomes for onychomycosis patients being treated with a topical antifungal. A comprehensive overview of the benefits of oral therapies will be necessary to counter the inevitable search for side effects and potentially course-limiting bad information. First, it is important to emphasize that this will be a long-term solution, aiming to reverse a disease process that did not develop overnight and in many cases has been self-diagnosed, diagnosed but not cultured, and as a result progressively worse for several years [52]. This is a condition where slow gains should be celebrated. This is a marathon and not a race, but this starts from the confirmation of the diagnosis. While it could take many months for the diseased nail to grow out completely, improvements are likely to be noticed as early as 12 weeks, especially in females and those with more recent disease. Optimizing concomitant management strategies is also critical to keeping the disease under control. This includes proper nail care, treatment of recurrent tinea pedis and eradication of the nail reservoir of dermatophytes, use of nail polish and potential issues with therapies, and the potential for recurrence with non-adherence to the extent of the treatment course. Finally, the consequences of not treating this disease has to be part of routine counseling. The discussion of other nails potentially getting infected, dissemination to other body parts, and concomitant infections has to also be had with the patients. In other studies, it has been recognized that patients with long-standing disease had more non-target toenails affected [52,53].

## 11. Conclusions

The goals of treating onychomycosis are a mycological cure and a normal appearing nail. The recent development of topical antifungals has been successful at improving the nail permeation and efficacy. Incomplete treatment of onychomycosis provides an environment favorable to the development of “antifungal resistance”. New topical agents and device-based therapies expand the therapeutic options. In addition, combination therapy may also improve the overall efficacy of antifungal treatments.

There is a clear need for further research, particularly randomized controlled trials, investigating the eradication and cure of onychomycosis. However, it is an exciting time with the advent of the new promising topical antifungals, and lasers and light systems that are relatively noninvasive treatment options; and thus, are on the forefront of fulfilling this critical need.
